# Quantitative Assessment of Experimental Ocular Inflammatory Disease

**DOI:** 10.3389/fimmu.2021.630022

**Published:** 2021-06-18

**Authors:** Lydia J. Bradley, Amy Ward, Madeleine C. Y. Hsue, Jian Liu, David A. Copland, Andrew D. Dick, Lindsay B. Nicholson

**Affiliations:** ^1^ School of Cellular and Molecular Medicine, University of Bristol, Bristol, United Kingdom; ^2^ Academic Unit of Ophthalmology, Translational Health Sciences, University of Bristol, Bristol, United Kingdom; ^3^ University College London, Institute of Ophthalmology, London, United Kingdom

**Keywords:** uveitis, EAU, OCT, image processing, automated analysis

## Abstract

Ocular inflammation imposes a high medical burden on patients and substantial costs on the health-care systems that mange these often chronic and debilitating diseases. Many clinical phenotypes are recognized and classifying the severity of inflammation in an eye with uveitis is an ongoing challenge. With the widespread application of optical coherence tomography in the clinic has come the impetus for more robust methods to compare disease between different patients and different treatment centers. Models can recapitulate many of the features seen in the clinic, but until recently the quality of imaging available has lagged that applied in humans. In the model experimental autoimmune uveitis (EAU), we highlight three linked clinical states that produce retinal vulnerability to inflammation, all different from healthy tissue, but distinct from each other. Deploying longitudinal, multimodal imaging approaches can be coupled to analysis in the tissue of changes in architecture, cell content and function. This can enrich our understanding of pathology, increase the sensitivity with which the impacts of therapeutic interventions are assessed and address questions of tissue regeneration and repair. Modern image processing, including the application of artificial intelligence, in the context of such models of disease can lay a foundation for new approaches to monitoring tissue health.

## Introduction

Ocular inflammation is an important medical concern with a wide range of manifestations from the easily treatable to sight threatening. It arises both as an ocular specific condition and in association with systemic disease and it manifests as more than 30 defined uveitic phenotypes. The pathogenesis is complex and multifactorial and there is a lively debate as to the relative contribution of subclinical infection, autoinflammation and autoimmunity (1, 2). Conventional approaches to imaging do not distinguish between these different causes.

Animal models of uveitis are often autoimmune (e.g. experimental autoimmune uveitis; EAU), inspired in the mouse by early work identifying susceptible strains (3, 4) and used widely to probe important aspects of immune function including tolerance (5, 6), regulation (7, 8), microbiome (9), lymphocyte dynamics (10) and macrophage/monocyte function (11). But other models of ocular inflammation are also important, including endotoxin induced uveitis (EIU) (12–14) and primed mycobacterial uveitis (PMU) (15). Ocular infectious disease can also be studied and has proven to be an informative model of inflammation (16–18).

Over the last 15 years, techniques for imaging the mouse retina have advanced substantially, first with fundal photography, acquired by topical endoscopic fundal imaging (TEFI) (19–21) facilitating clinical grading by individuals blinded to the origin of the images. Then followed by adaptation of clinical tools (12) and development of the Micron system for imaging rodent eyes (Phoenix technologies, CA). These advances have made acquisition of experimental image data more accessible and routine (12, 22–24). The application of optical coherence tomography (OCT) to the mouse eye adds new information on changes deep in the tissue. The eye offers unique advantages for imaging studies of the autoimmune process in a target tissue, permitting serial assessment, and sophisticated quantification of different parameters of inflammation that go beyond more general clinical scores used in models such as experimental autoimmune encephalomyelitis.

Advances in image processing that have been developed in patient populations can also find application in experimental studies. There is potential for automatic segmentation of structures (in which the boundaries between, for example, different layers of the retina are identified in an unsupervised process), quantification of infiltration and disease classification by machine learning, which can be used to support unsupervised clinical assessment (25, 26). This is seen in the recent application of deep learning to EAU (27). Alternative powerful technologies are also available; using bioluminescent reporters, can delineate sequential cell population specific patterns of infiltration (28, 29), and multi-optical imaging approaches can produce data on phenotype and the spatial relationship between different cell types (30). Objective measurements, that provide a more granular multi-modal analysis of the state of the tissue, can then form the basis for quantifying the impact of treatment on ocular disease not limited to a single time-point but integrated across a longer disease course.

## Ocular Tissue and Inflammation

**Figure 1:**
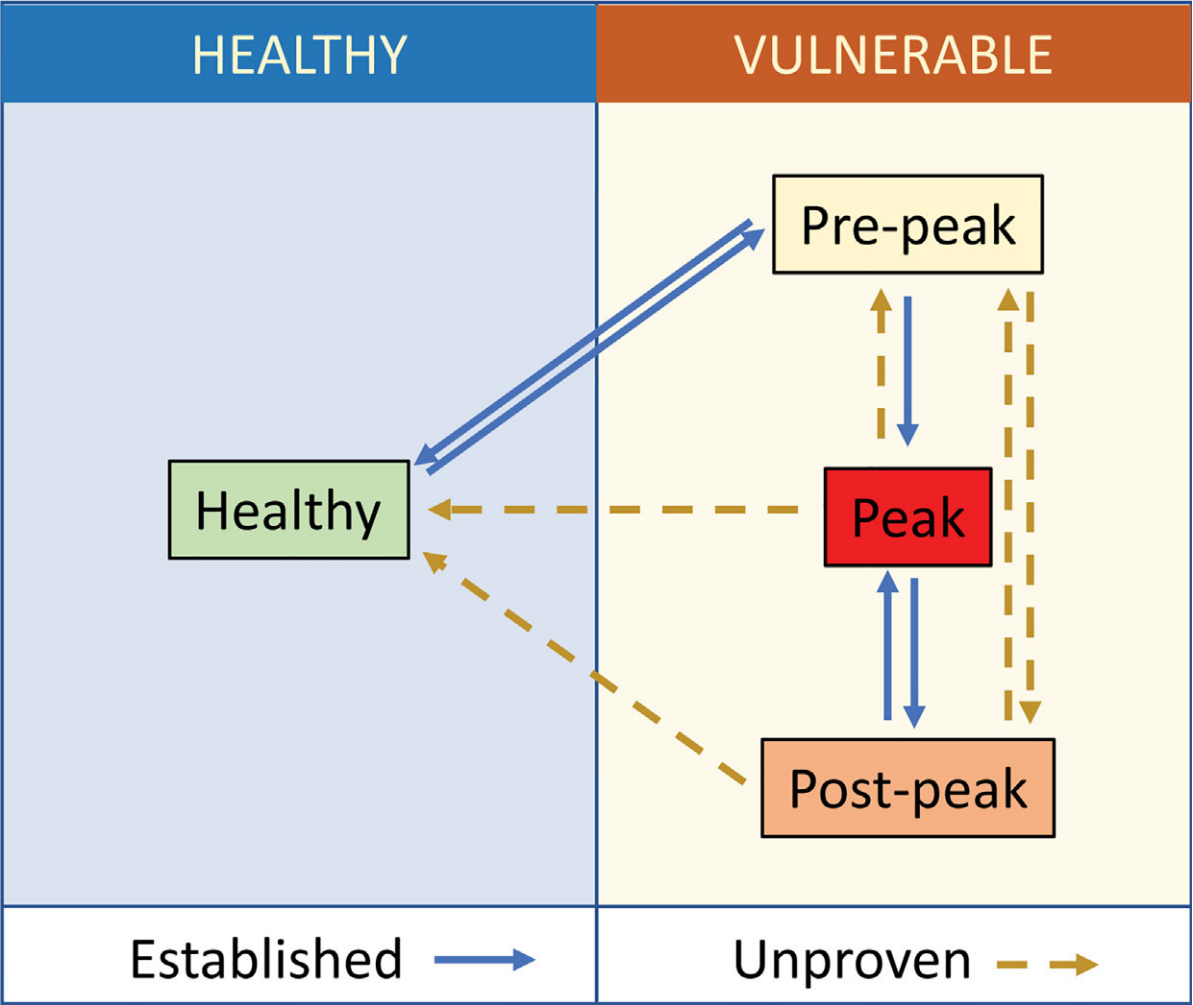
Tissue states in ocular inflammation. Healthy ocular tissue is ‘immune-privileged’ and under low-level immunosurveillance. Specific (ocular antigen driven) and non-specific (extra-ocular inflammation) stimuli disturb this homeostasis and increase interactions across the blood retinal barrier making the tissue more vulnerable to the development of disease. In uveitis following active immunization, this starts with the prodrome (8), which can resolve back to the healthy state. When the prodrome progresses to clinical EAU in immunocompetent animals, there is an influx of cells to a maximum (peak) followed by a reduction in immune cell content, which does not return to base line. The post-peak (in EAU described as secondary regulation) is distinguished from the pre-peak by changes in the relative proportion of different lymphocyte populations (CD4 T regulatory cells, CD8 T resident memory cells). There is currently no evidence that disease proceeds directly from pre-peak to post-peak, nor that eyes that have reached peak disease ever return to the normal healthy state.

EAU is often studied with a focus on the acute inflammation that occurs with the explosive influx of immune cells that flood into the tissue in the first wave of clinical disease. But it has been apparent for a number of years (31, 32) that it can also be used to develop insights into the processes of persistent disease and tissue remodeling. For example, memory cells that reside in the bone marrow are implicated in chronic retinal degeneration (33) and persistent inflammation can lead to retinal angiogenesis (34). In both mouse (35) and human (36), chronic disease can drive the development of ectopic lymphoid like structures and is accompanied by changes in the other lymphocyte populations and vascular remodeling (10, 34). The ocular tissue can therefore exist in a minimum of four well demarcated states (**Figure 1**).

Healthy tissue resists insult and maintains normal visual function. In the EAU model, there are a minimum of three non-healthy states, which correlate with changes in immune cell content and vascular function (31). Vulnerable tissue may be in the prodromal phase of EAU, at peak of disease, with active infiltration by many different leukocytes, or vulnerable but to a greater or lesser extent recovered, which state is described as post-peak. It is possible to observe experimentally that the pre-peak state can resolve to a state of health, or progress to peak disease. Tissue can reach peak disease from either the pre-peak state or as a relapse from the post-peak state (37). But it is unknown whether from peak or post-peak, tissue can ever return to a healthy state. In the broader context, a useful framework for these changes is found in the extensive literature describing the development and resolution of inflammation, but here too, the question of active resolution in the tissue and the mechanisms by which it occurs remains controversial (38). While this review focuses studies in the eye, it is evident that other diseases and disease models, such as arthritis, can be fitted into a similar framework (39).

One essential tool for advancing understanding of these different tissue states is a rigorous method of clinical assessment that separates healthy tissue from the vulnerable and that also distinguishes between different states of the vulnerable tissue. Such a scheme could then complement studies describing gene expression in different forms of ocular inflammation (13, 40). Recent advances in the range and quality of techniques that can be applied to quantify ocular inflammatory disease make such objective and transferrable assessments increasingly feasible.

## Assessment of Ocular Inflammation

The measurement of inflammatory activity is a core objective for clinical studies of uveitis and has inspired work that seeks to improve its ability to discriminate between lower levels of disease as well as improving its sensitivity (41). Progress in this area can also inform animal studies.

### Clinical Scoring

**Figure 2:**
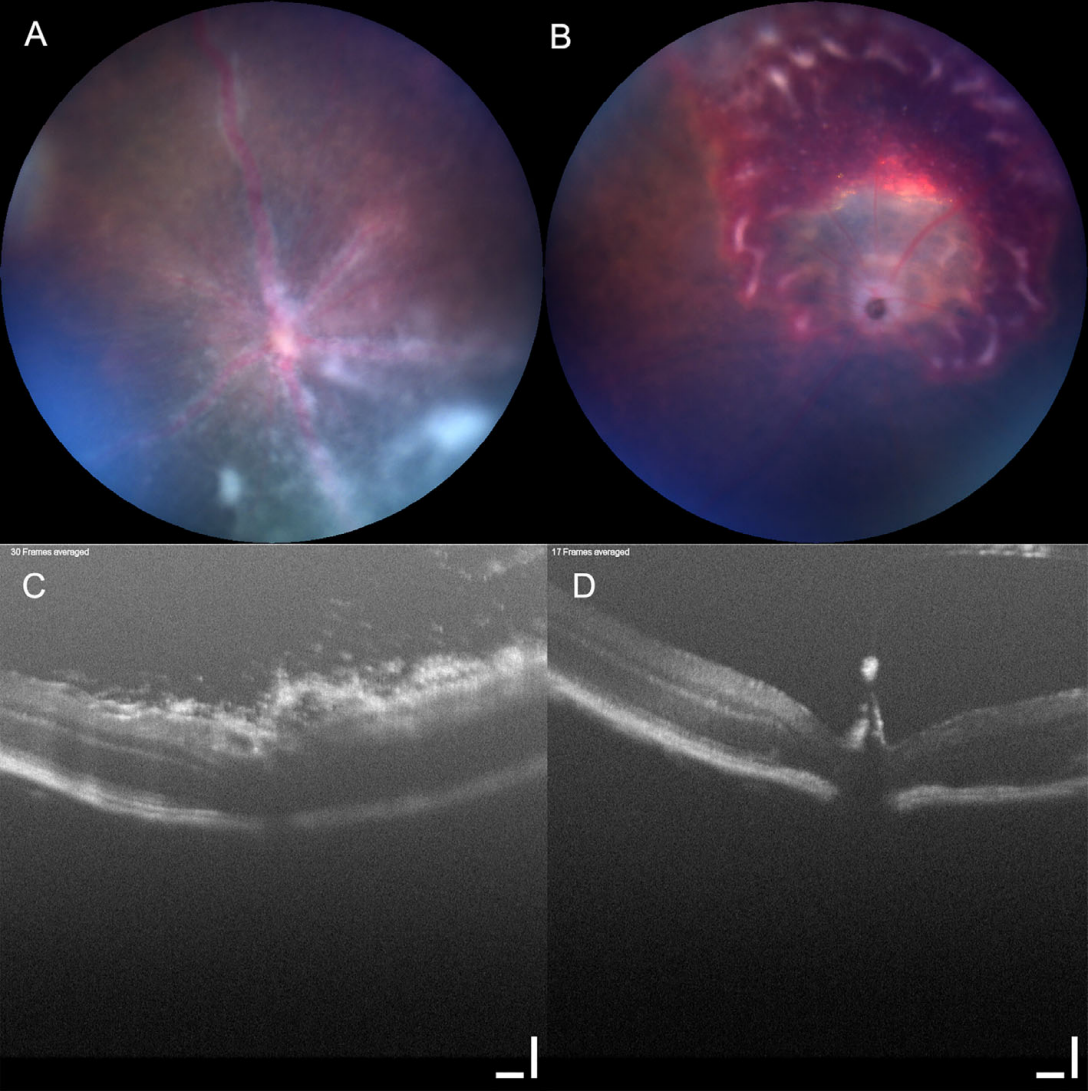
Clinical score can be insensitive to underlying pathology. Mouse eyes imaged using Micron IV with OCT (Phoenix technology group, CA). Two mouse eyes **(A, C)** and **(B, D)** imaged using Micron IV (Phoenix technology group, CA) and assessed by fundal photography **(A, B)** and OCT **(C, D)**. Retinal photographs scored in a set of images by an observer blinded to the treatment groups, both received the same summary clinical score. Scale bar 100 µm.

In human eye disease, improvements in imaging have driven diagnostic sensitivity and specificity (42, 43). Scoring systems serve as tools for categorizing disease activity into ordinal groups and as a convenient measure of clinical outcome and directional change. The first aqueous and vitreous inflammation scoring systems based on ophthalmic observation of cell counts in patients were published in 1959 (44, 45), but consensus recommendations did not emerge until 2005, under the umbrella of the Standardization of Uveitis Nomenclature (SUN) workshop (46). For some diseases, for example Behçet’s disease, specific scoring systems have proven useful is assessing treatment response (47). It is a recognized concern with scoring systems that there is a tension between precision and simplicity. Levels of interobserver agreement remain modest and non-linearity in the scaling can lead to poor resolution of differences in disease especially at lower levels of inflammation (48–50). The use of digital images, where biological data is quantified as pixel values, expands the possibilities for analysis by computer imaging (25) for example for automated grading of vitreous haze (51). Scoring of clinical disease in EAU has evolved from early approaches using slit-lamp aided visualization and semi-quantitative histological scoring to more sophisticated scoring approaches based on blinded assessment of fundal photographs (20, 21, 23, 52, 53) and most recently using machine learning. Scoring can be on a simple ordinal scale (0–4) or can categorize disease into three indicators of inflammation and one of structural damage with inflammation and structural damage reported independently or as a summary score (0–5) calculated as the total or average score for the eye (10, 21, 54) (**Table 1**). When applied as a summary score, this approach can be insensitive to differences in aspects of the underlying pathology, for example in **Figure 2**, the two images, although clearly different, received the same summary clinical score.

**Table 1:** Scheme for scoring clinical ocular inflammation.

Score	Optic disc	Retinal vessels	Retinal tissue infiltration	Structural damage
1	Minimal inflammation	Cuffing: 1–4 mild	1–4 small lesions or 1 linear lesion	Retinal lesions or retinal atrophy involving 1/4 to 3/4 of retinal area
2	Mild inflammation	Cuffing: >4 mild or 1–3 moderate	5–10 small lesions or 2–3 linear lesions	Panretinal atrophy with multiple small lesions (scars) or ≤3 linear lesions (scars)
3	Moderate inflammation	Cuffing: >3 moderate	>10 small lesions or >3 linear lesions	Pan-retinal atrophy with >3 linear lesions or confluent lesions (scars)
4	Severe inflammation	Cuffing: >1 severe	Linear lesion confluent	Retinal detachment with folding
5	Not visible (white-out or extreme detachment)	Not visible (white-out or extreme detachment)	Not visible (white-out or extreme detachment)	Not visible (white-out or extreme detachment)

A blinded observer assigns scores to retinal photographs for changes that relate to inflammation of the optic disc, retinal vessels and retinal tissue and a score for structural damage. These scores can then be summed independently (score of 0-20) or given as a summary score of the average of all features (score of 0-5) (10, 21, 54).

Complementing photography is optical coherence tomography (OCT). Developed in the 1990s (55, 56) it has rapidly become the state of the art for non-invasive retinal imaging. OCT is an interferometric technique providing depth resolved cross sectional images of the retina, known as B-scans. In normal eyes the vitreous is optically transparent, retinal layers show different degrees of backscatter, and in humans the RPE is one of the most hyper-reflective layers. Modern OCT in humans can also go some way to visualizing the choroid beneath the RPE (57). OCT can resolve retinal substructure and its vasculature, can be important in the diagnosis and image guided management of human uveitis and can capture changes in the state of the tissue through time in EAU (12, 24, 58, 59).

### Ocular Tissue Analysis

In contrast to the wealth of sophisticated imaging that can be directed at the human eye in uveitis, access to human tissue is severely limited. Enucleation of the globe in uveitis is rare and is usually from individuals with long-standing disease (36). But in the EAU model, histology was the first accepted standard for disease assessment (60–62). Immunohistochemistry and immunofluorescence of retinal tissue revealed the profound structural disruption that accompanies acute inflammation, and was used, for example, to show how macrophages reciprocally alter their expression of CD68 and arginase-1 during the persistent (post-peak) phase of uveitis (34). For higher dimensional analysis of cell infiltrate, investigators have used multiparameter flow cytometry which can quantify many different cell populations (8, 63, 64). Sampling the cell infiltrate at different time points has been instrumental in demonstrating important changes in the relative frequencies of CD4 T regulatory cells (65) and CD8 cells (10). In EAU this is strong evidence that at the cellular level as well as in serial imaging studies, the tissue and the immune infiltrate change and adapt through time. Developing improved quantitative methods to assess tissue health in EAU offers more sensitive and specific approaches to analyze the impact of therapies for autoimmunity and inflammation.

### Quantitative Assessment of EAU

Using formal criteria, EAU can be assessed semi-quantitatively, but interobserver disagreement and subjectivity limits the usefulness of direct comparison between results from different labs and even individual researchers (21). As with human clinical graders, experience is required to achieve the highest levels of interobserver agreement (66). Employing contemporary technology has the capacity to improve on these limitations. In addition, in EAU as in other medical images, these can be annotated, with the results of end point tissue analysis added to the meta-data associated with the image. This enriches their interpretation and provides a resource that can be applied to other studies. Pooling data from animal cohorts at selected timepoints runs the risk of obscuring subtle patterns, and overweighting the importance of the certain trends. This can be countered by the use of analysis that exploits modern image processing, with its scope for a higher degree of quantitation (66–68). A critical element of complementary analysis is therefore the use of non-invasive techniques and computational means to maximize information retrieved from the data.

Fundus photography, for example obtained by TEFI, correlates well with disease scores from histopathological analysis (20) but the images produce a 2D projection of 3D semi-transparent biological tissue. Spatial information is only available in two dimensions and artifacts are introduced by flattening depth information onto a plane. More accurate measures of infiltrate, oedema and structural changes, that are important manifestations of disease, can be obtained with OCT (24, 59). Because OCT produces a depth profile of different features, it can be more sensitive than 2D fundus imaging in monitoring the appearance and development of pathological changes. In particular, cross sectional images are more sensitive to early disease because they can visualize small amounts of infiltrate around the optic nerve, and measure changes in optic nerve diameter and retinal thickness due to inflammatory oedema (23, 59, 66, 68).

### Aqueous and Vitreous Assessment

A defining characteristic of uveitis is cellular infiltrate, and grading is an important quantitative metric in preclinical animal model research. In human disease, anterior uveitis produces ‘flare’ which can be categorized by laser flare photometry and which correlates well with conventional clinical grading (69, 70) while in the vitreous, ‘haze’ is an accepted and clinically validated proxy for inflammatory status in patients (51). Moreover, these changes have a marked impact on visual acuity in humans and so are biologically and clinically relevant outcome measures (50).

In OCT, cells in either chamber appear as hyperreflective dots, whose profile is a function of many variables (71–73). Cells and exudate incrementally reduce the optical transparency of the ocular media leading to the aqueous and vitreous becoming inhomogeneous as disease severity increases. These changes reduce the contrast of object boundaries and the results of qualitative or quantitative image analysis lose precision.

Because of difficulty in imaging the anterior chamber of small eyes, literature for OCT based cell counting in these models is relatively sparse (74). However, automated counts of absolute cell numbers have been obtained with excellent correspondence to manual image counts. This approach has been developed into a fully automated pipeline for cell counting in volumetric OCT images, achieving 98% congruence to manual slit lamp counts. Importantly, the subjective manual element of the segmentation step was eliminated. The automated segmentation step involved removal of anatomical structures connected to image boundaries (75). Compared with counts from histological sections, OCT tended to undercount, which was attributed to insensitivity to cell clumps, sediments and exclusion of the extremities of the iris interface (74). It may also be contributory that histology is unaffected by overlying opacities, whereas OCT is vulnerable to signal degradation. However, histology introduces artifacts and postmortem changes that themselves affect tissue measurement (74).

Loss of precision becomes more evident when imaging the vitreous, where the optical pathway traverses deeper through affected media. Further complicating the analysis of the rodent vitreous, is the anatomical vestige of the hyaloid artery (71, 76), protruding upwards from the optic disc towards the lens. It confuses the vitreoretinal boundary and can appear somewhat discontinuous, with hyperreflective regions that are subjectively indistinguishable from cell clusters.

Automated counting algorithms usually require a preceding segmentation step, that defines a boundary for the area or volume of interest. Variations in signal quality and the ambiguity of discontinuous image features frustrate the development of accurate, fully automated methods of rodent image segmentation and analysis. Quantification of changes in the vitreous has largely been restricted to human images, and global signal parameters, as opposed to absolute cell counts.

To account for signal strength variations in human OCT images, the average intensity of the segmented vitreous compartment can be indexed relative to a hyperreflective reference layer such as the RPE, providing a relative intensity ratio. These ratios correlate moderately with clinical vitreous haze scores, along with other surrogates of disease such as retinal thickness (72, 77). This process has been fully automated using rule-based algorithms for segmentation, reducing subjectivity. The same operation was also performed using a textural descriptor of the vitreous, which was marginally better correlated to clinical scores than vitreous intensity (73). These operations were performed on 2D datasets, obtaining an averaged intensity ratio based on several B-scans and data analyzed in 3D may potentially offer further improvements.

Since the scan region is much smaller than the ocular globe, one consideration is the selection of a representative and informative region of interest (ROI) that must be equivalent between scans and subjects. Within human images, landmarks such as the macula can be located automatically and used as a central anchor point for region boundary positioning (73). In rodents, the optic disc is an obvious landmark choice, but the presence of the hyaloid remnant, particularly in severely diseased eyes warrants additional steps to remove its influence. Recently, an automated method of quantifying vitreous inflammation in clinical fundus photographs has been suggested (50, 51).

### Retinal Layers

**Figure 3:**
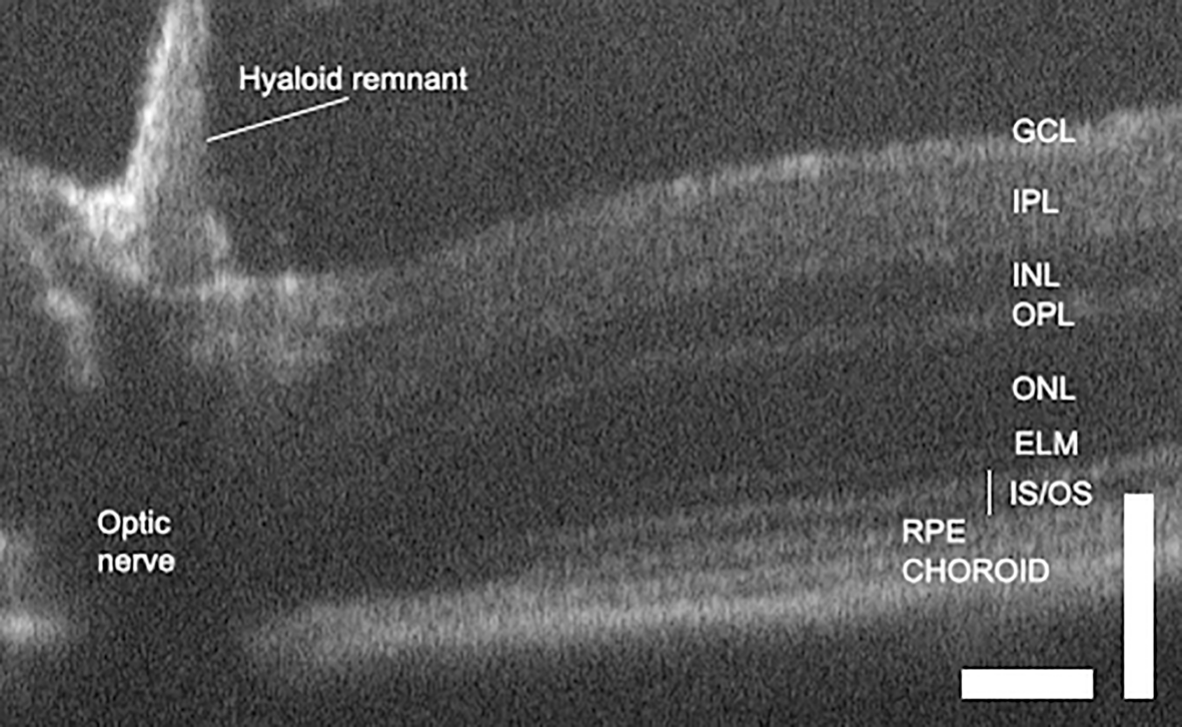
OCT of the normal mouse retina delineates layers and allows retinal dimensions to be quantified. Scale bars are 100 microns and illustrate differences in axial and lateral resolution. GCL, ganglion cell layer; IPL, inner plexiform layer; INL, inner nuclear layer; OPL, outer plexiform layer; ONL, outer nuclear layer; ELM, external limiting membrane; IS/OS, inner and outer segments; RPE, retinal pigment epithelium (68).

OCT of the healthy retina produces good definition of the different layers of light sensitive tissue. In uveitis it can resolve and localize lesions and pathologies, and identify vasodilation and perivascular exudate (24, 59). Standard clinical OCT has an axial resolution of less than 4 microns, which can produce images with near histological detail. Thickness is ascertained from OCT images by measuring the distance between two boundaries of choice (**Figure 3**). Before measurements can be taken, the layers must be defined.

Techniques for segmentation to define different retinal layers have progressed through manual, semi-automated and fully automated protocols, with work on human data leading rodent OCT imaging. Both rule-based algorithms and learner-based approaches have been applied to the problem and new approaches are under active investigation. Retinal thickness can be measured by OCT absolutely, using assumptions such as an average tissue refractive index (78), or by fold change compared to pre-disease measurements (66). Both are in high agreement with histological measurements (24, 59, 66, 78, 79). Several schemes exist for displaying changes in thickness. One that is commonly used shows thickness at different distances from the optic nerve head (**Supplementary Figure 1**).

Rule-based methods execute a pre-programmed set of instructions, designed with the expected properties of the image and the desired features in mind. Many image properties can be analyzed, including intensity variation, geometric contours and texture (80–84). The number of segmented layers defined varies between four and nine, and depends on the approach, with the most successful techniques to date being learner models (26, 80, 85–88)

OCT offers the potential of assessing layer deformation without the artefacts that can be introduced by tissue fixation, sectioning and staining (89). Mechanical deformation can also introduce ambiguous artifacts, with likeness to retinal detachments (78), and congenital abnormalities in the retina may also confound the definition of anatomical normality (90). The literature pertaining to automated quantitation of retinal structure is more extensive than that related to infiltrate, because retinal layer changes are associated with a wide variety of ocular diseases (91, 92). The laminated reflectance profile of the retina’s architecture also lends itself to image segmentation and the measurement of quantitative indices such as layer thickness and geometric descriptors. Protocols for automatic layer segmentation developed for human studies have been tested in different mouse strains. These performed well when assessing the inner retinal layers, but were less successful in defining the murine RPE, whose location displaced distally into the sclera (68).

Longitudinal studies of retinal thickness have revealed details about the kinetics of disease progression, with respect to other important manifestations of pathology (59, 66). In the pre-peak to peak phase of disease, retinal thickness increases rapidly due to inflammatory oedema, correlating with inflammatory infiltrate, measured longitudinally by OCT and confirmed by histology (66, 78). In the post-peak resolution phase, the clearance of exudate reveals features on OCT with greater clarity, such as infiltrate, photoreceptor atrophy, retinal folds and choroiditis (59). Photoreceptor damage persists beyond the peak phase of disease as retinal oedema is slower to resolve than inflammatory infiltrate. When the swelling does subside, the retina thins to below pre-disease levels because of photoreceptor loss. OCT confirms that neither infiltrate or retinal thickness returns to baseline in late disease or even after resolution is complete (20, 59, 78). Therefore, quantitative directional changes and relative rates of change between retinal thickness and inflammatory infiltrate can provide an additional metric for disease activity.

In severe uveitis, retinal layers are obscured by opacification of the vitreous and aqueous due to infiltrate and proteinaceous exudate (59) which presents a challenge for scoring systems, that must be robust to substantial signal variation and may need to incorporate metrics of opacity into the model as proxies of inflammation.

### Vasculature

Important changes in the vasculature occur in uveitis, including ischemia, neovascularization and retinal/choroidal vasculitis (93). In disease models these are assessed less commonly than structural changes, but as in humans they are often interrogated by angiography. Confocal scanning laser ophthalmoscopy (SLO) can be coupled to fundus fluorescein angiography (FFA) to quantify vessel diameter and leakage in EAU. When average vascular dilation was measured immediately prior to sacrifice and histology, major vessel diameter was well correlated with retina-choroid thickness and with clinical and histological scores. This indicated that inflammatory vasodilation of superficial vasculature was a novel measure of EAU severity (66). Complementary to dye-based angiography are OCT based methodologies. Vascular dilation and perivascular exudate attributed to retinal vasculitis can be localized to specific retinal layers during the course of EAU (23, 59) and OCT has been used for imaging vasculature disturbances, such as choroiditis and retinal vasculitis (43). Blood flow can be visualized and depth resolved (94) using OCT angiography (OCTA) and this has been used to assess retinal microvascular changes (95, 96).

Many methods of segmenting retinal blood vessels from fundus photographs have been published (97). A much smaller number of approaches have been successfully devised using OCT images, which include the use of multimodal imaging (corresponding fundus photographs) and learner models (98, 99). In humans, segmentation of fine capillary networks has been achieved in OCTA enface images (100) while in mice segmentation of retinal vasculature using OCTA has been reported for longitudinal monitoring of angiogenesis (101). Current advances applying deep learning to vessel segmentation continue to improve the performance of these methods and this has been helped by the public access to data sets (102).

### Functional

As EAU progresses, electroretinogram (ERG) amplitudes change. There is a dramatic reduction in function (a and b wave), that accompanies early disease (103), presenting before morphologic changes. These findings indicate that functional loss could be mediated by inflammation rather than just physical damage, and that retinal function is potentially a sensitive early indicator (59, 66). However, photoreceptor damage continues while inflammation is receding and in the post-peak phase, ERG amplitudes are correlated with OCT measures of retinal thickness. As swelling diminishes, photoreceptor atrophy becomes apparent and results in an overall retinal thinning compared to baseline. Neither retinal thickness nor functionality ever fully recover (59, 103).

Taken together, multimodal quantitative measures can provide information on perceptually subtle, but biologically significant changes whose quantification would aid clinical grading and pre-clinical research.

## Examples of Multimodal Measurement

**Figure 4:**
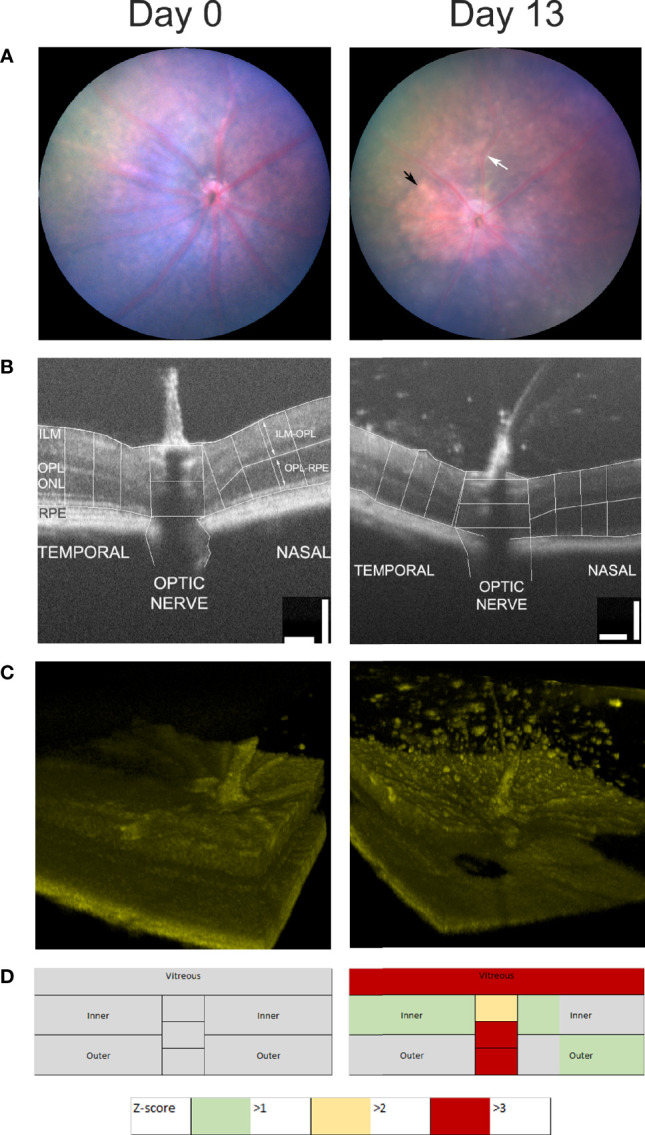
Multimodal analysis of EAU. Mouse eyes were imaged at day 0 and day 13 after the induction of EAU and one representative image of the same eye is shown **(A–C)**. Clinical disease can be assessed by photography **(A)**, measurements of retinal thickness and optic nerve diameter at three points from the temporal, nasal and optic nerve regions of the OCT B-scans **(B)**, 3D-reconstuction of retinal infiltrate **(C)** and summary data of retinal scores from all groups **(D)**. Summary scores are assembled from unsupervised quantitative assessment of vitreal involvement, manual segmentation and measurement of inner and outer layer thickness and optic nerve diameter transformed and represented as Z-scores.

A multimodal approach to assessing uveitis is outlined in **Figure 4**. EAU was induced by the transfer of pathogenic autoantigen reactive T cells. Sequential imaging of all eyes was carried out by fundal photography and OCT. B-scans were segmented manually and measured by an observer blinded to treatment conditions. Measurements of retinal thickness were made at baseline from all eyes (n=11) and these were compared as a Z-score expressing the magnitude of change in thickness on day 13 color coded as the number of standard deviations from baseline (**Figure 4D**). **Figures 4A–C** shows images from a representative single eye at baseline and day 13. The retinal photographs (**Figure 4A**) show that at day 13 there is an enlarged optic nerve, sheathing of the vessels due to cell infiltration (white arrow) and infiltrates in the tissue (black arrow). B-scans (**Figure 4B**) through the optic nerve, were assembled from multiple averaged frames and are displayed with the accompanying 100 micron scale bars that were used to generate measurements of the retinal thickness following manual segmentation using ImageJ (104). At day 13 it is easy to see objects in the vitreous around the optic nerve. The 3D image (**Figure 4C**) is prepared from 512 sequential B scans, processed using code in MATLAB (Natick, Massachusetts: The MathWorks Inc) and ImageJ (26) adapted for use with murine images and rendered using ImageJ (1.53 3D viewer plugin). These pictures give a better appreciation of the spatial distribution of the vitreal infiltrate and can be used to make a semi-quantitative estimate of the degree of vitreal infiltration.

**Figure 5:**
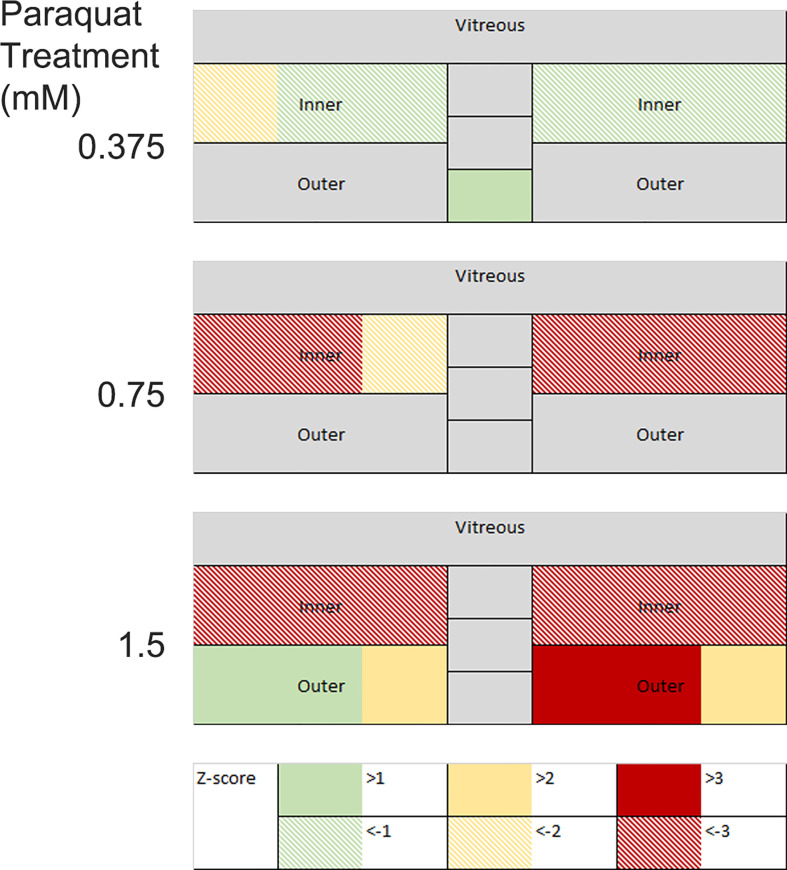
Changes in retinal thickness in mouse eyes following intra-vitreal paraquat instillation were measured on day 10. Images were visualized by OCT, manually segmented, and measured at three points in the temporal, nasal, and optic nerve regions. Measurements are expressed as positive and negative Z-scores relative to a PBS injected control group. Changes in the inner and outer layers are decoupled.

Following changes in disease scores through time, it is useful to display the aggregate data from the multiple images, and this has been used to produce a color-coded map of the retina, with changes normalized to baseline scans (usually on day 0) and scaled by Z-score. Retinal maps are also useful when comparing the pattern of pathological change between different disease models. For example, compare **Figure 4D**, which shows that at day 13 the major impact of uveitis is found in the vitreous and the optic nerve with **Figure 5** which shows the does dependent effect of intra-vitreal instillation of paraquat, a model of oxidative stress, in C57BL/6 mice. This induces neuronal degeneration which varies with stain (105) and in this case particularly impacts the inner retina, seen as a negative Z-score increasing in magnitude with dose. But quantitative analysis also reveals that at higher concentrations of paraquat, this is accompanied by an expansion of the outer segments, due to inflammation. This finding, using multimodal analysis is in agreement with a previous report showing more pronounced TUNEL-positive cells in the inner retina than in the outer retina of C57BL/6 mice treated intravitreally with paraquat (105).

### Opportunities for Automation

Machine learning has made an impact in human clinical care in recent years because of its ability to reach expert-level diagnosis. The automated analysis of ocular disease has led the way in carrying these methodologies into the clinic, but they have been less extensively utilized in disease models (106, 107).

Images are inherently data rich because in theory each pixel can be regarded as a separate input parameter (106). This offers opportunities for uncovering novel aspects of pathological processes but also challenges, especially in assembling well annotated data sets that are large enough to avoid overparameterization when they are used to train classification algorithms in a machine learning framework. Advances in predictive statistical methods may in time alleviate the need for such extensive input data. One helpful approach, applied in OCT, is decoupling the methods for segmentation from artificial intelligence driven disease classification (108). This moves practice towards device-independent representation of the disease process, which may aid in comparison between studies carried out by different investigators.

Recently the field has advanced with the application of a deep learning model to analyze photographs of the retinas of mice with EAU. Using a data set of images that was extended by data augmentation, disease images were divided into three categories and by applying deep learning methods (convolutional neural networks) the overall performance assessed by area under the receiver operating characteristic curve (AUC) when the model was applied to an external dataset of 33 images was approximately 0.90 (27).

Another area of opportunity in multi-modal ocular imaging is the fusion of information from different modalities such as fundal photography and OCT (109, 110). Image fusion aims to yield a more complete, accurate and efficient account of an object by combining different visualizations together. Integrating this methodology into the assessment of experimental clinical disease will inform our ability to distinguish between different states of tissue health (**Figure 1**).

## Conclusion

Persistent ocular inflammation is a significant and challenging clinical entity that is associated with long term changes in the retina and serious sight threatening complications (111). Experimental models of non-infectious and infectious ocular inflammation have been widely and successfully deployed. But fundamental insights regarding how tissue homeostasis is perturbed and how it might be restored are still needed (112). Such concerns are important in a much broader context than uveitis. Restoring complex tissues, damaged by persistent inflammation, to normal physiological function will have wide application. Multimodal and quantitative imaging of the eye, in an experimental context, has potential to advance our understanding of the kinetics, cell biology, transcriptomic and proteomic architecture of how this multifactorial process is regulated. By providing non-invasive techniques to probe the underlaying nature of the tissue, there is an opportunity for a more precise and comprehensive discrimination between different states that can be used to stratify information gleaned from detailed examination of the transcriptome and microbiome, multiparameter flow cytometry and proteomics.

## Author Contributions

Literature survey and drafting of original manuscript and figures (LB, AW, LN). Preparation and provision of data (AW, MH, JL, DC). All authors contributed to the article and approved the submitted version.

## Conflict of Interest

The authors declare that the research was conducted in the absence of any commercial or financial relationships that could be construed as a potential conflict of interest.
