# Supplementary figures and images for: Quantitative Assessment of Experimental Ocular Inflammatory Disease
Source: Front Immunol. 2021 Jun 18;12:630022. doi: 10.3389/fimmu.2021.630022 (PMC8250853; doi:10.3389/fimmu.2021.630022)

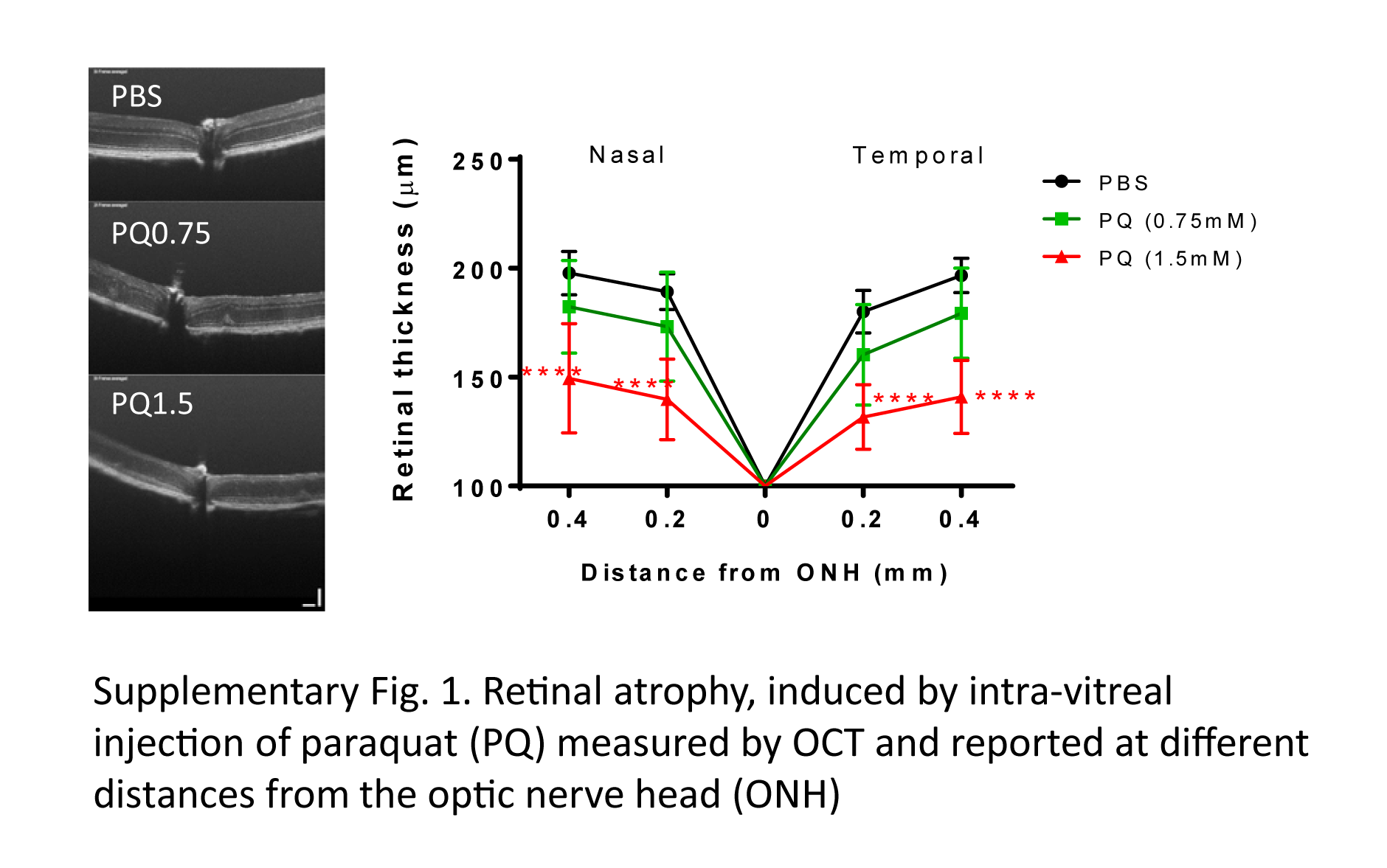

Supplement: Supplementary file 1 [file Image_1.tif]
